# Metabolite Profiling Identified Methylerythritol Cyclodiphosphate Efflux as a Limiting Step in Microbial Isoprenoid Production

**DOI:** 10.1371/journal.pone.0047513

**Published:** 2012-11-02

**Authors:** Kang Zhou, Ruiyang Zou, Gregory Stephanopoulos, Heng-Phon Too

**Affiliations:** 1 Chemical and Pharmaceutical Engineering, Singapore-MIT Alliance, Singapore; 2 Department of Biochemistry, National University of Singapore, Singapore; 3 Department of Chemical Engineering, Massachusetts Institute of Technology, Cambridge, Massachusetts, United States of America; Laurentian University, Canada

## Abstract

Isoprenoids are natural products that are all derived from isopentenyl diphosphate (IPP) and dimethylallyl diphosphate (DMAPP). These precursors are synthesized either by the mevalonate (MVA) pathway or the 1-Deoxy-D-Xylulose 5-Phosphate (DXP) pathway. Metabolic engineering of microbes has enabled overproduction of various isoprenoid products from the DXP pathway including lycopene, artemisinic acid, taxadiene and levopimaradiene. To date, there is no method to accurately measure all the DXP metabolic intermediates simultaneously so as to enable the identification of potential flux limiting steps. In this study, a solid phase extraction coupled with ultra performance liquid chromatography mass spectrometry (SPE UPLC-MS) method was developed. This method was used to measure the DXP intermediates in genetically engineered *E. coli*. Unexpectedly, methylerythritol cyclodiphosphate (MEC) was found to efflux when certain enzymes of the pathway were over-expressed, demonstrating the existence of a novel competing pathway branch in the DXP metabolism. Guided by these findings, ispG was overexpressed and was found to effectively reduce the efflux of MEC inside the cells, resulting in a significant increase in downstream isoprenoid production. This study demonstrated the necessity to quantify metabolites enabling the identification of a hitherto unrecognized pathway and provided useful insights into rational design in metabolic engineering.

## Introduction

Isoprenoids are a large family of compounds (more than 55,000) comprising numerous products used as fragrances, insecticides, nutraceuticals and pharmaceuticals [1], [2]. Supply of these molecules has been limited by scarce plant resources from which they were originally extracted. Production by chemical synthesis is uneconomical due to the complex structure of these products [1]. Microbial metabolic engineering has been intensively explored in the past decade for isoprenoid production and gram per liter production levels has been achieved for certain isoprenoid precursors [3].

**Figure 1:**
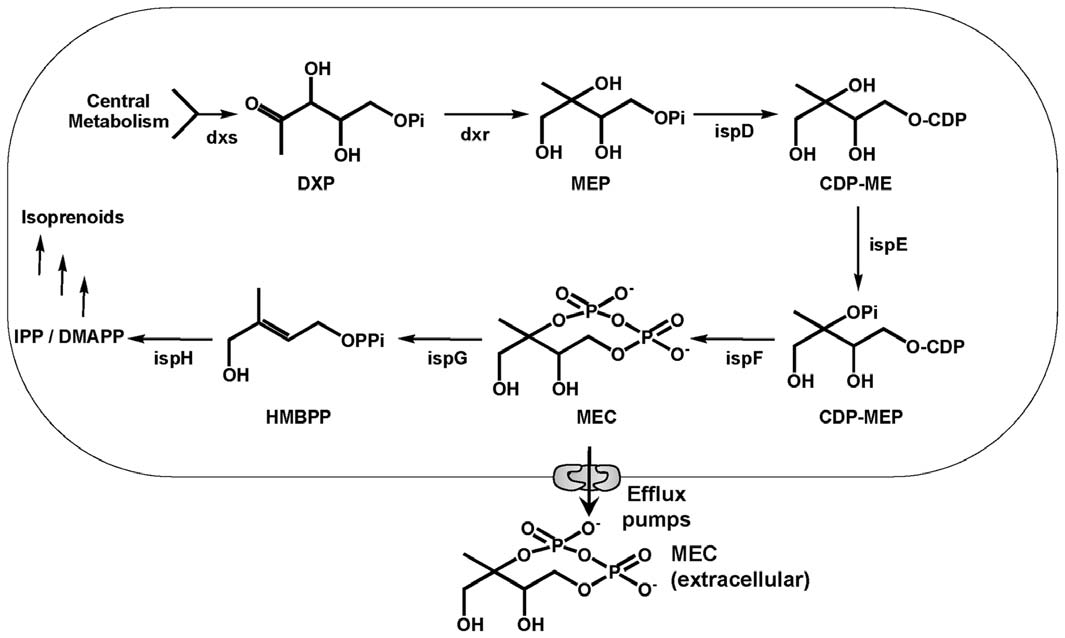
Metabolites and enzymes related to the 1-Deoxy-D-xylulose 5-Phosphate pathway.

Despite the structural diversity of isoprenoids, they are all derived from isopentenyl diphosphate (IPP) and dimethylallyl diphosphate (DMAPP). Both metabolites are synthesized either by the mevalonate (MVA) pathway or the 1-Deoxy-D-Xylulose 5-Phosphate (DXP) pathway (Figure 1) [4]. Engineering of the DXP pathway (also known as MEP pathway) in *Escherichia coli (E. coli)* results in significant increase in the biosynthesis of IPP/DMAPP [3], [5]. Four enzymes in the DXP pathway (dxs, idi, ispD and ispF) were identified combinatorially to be rate limiting [6] and overexpressions of the genes encoding these enzymes were empirically optimized to maximize isoprenoid yields. It is also known that excessive overexpression of these enzymes can inhibit isoprenoid production [3], [7], [8], [9], [10]. Recently, we demonstrated that strong overexpression of metabolic enzymes (dxs, idi, ispD and ispF) did not affect the expression of isoprenoid transcripts which was hypothesized to contribute to the decrease in lycopene production [11]. To better understand the mechanisms underlying the inverse correlation of lycopene production with overexpression of the DXP genes, a direct measurement of the metabolites is desirable.

Quantification of the metabolites of the heterologous mevalonate pathway in engineered *E. coli* allowed the identification of pathway limiting steps and generated rational gene targets for circumventing production inhibition [12]. Attempts have been made previously to measure methylerythritol cyclodiphosphate (MEC), a metabolite of the DXP pathway, in cell extracts using a semi-quantitative and labor intensive method involving ^31^P-NMR [13]. DMAPP and IPP, products of the DXP and MVA pathways, have also been previously quantified by mass-spectrometry [14], [15]. As yet, there is no direct method to simultaneously quantify all DXP pathway metabolic intermediates (Figure 1).

To address the unmet need of a method for the simultaneous quantification of DXP metabolites, we have developed an integrated preanalytical solid phase extraction (SPE) procedure with the use of ultra performance liquid chromatography mass spectrometry (UPLC-MS). The SPE procedure exploits the unique physiocochemical properties of the DXP intermediates to selectively enrich these compounds from biological samples. The performance of this SPE coupled UPLC-MS protocol was then demonstrated with synthetic standards in *E. coli* extracts, and was found to be superior in analytical sensitivity and ease-of-use. With the developed method, MEC was found to be unexpectedly effluxed from *E. coli*, by yet to be characterized mechanisms, which significantly reduced the production of isoprenoids. The overexpression of ispG was predictably found to result in lower MEC efflux and increased isoprenoid production. This study has developed invaluable tools for rational engineering of the DXP pathway for overproduction of isoprenoids.

## Results

### UPLC-MS analysis of the DXP pathway intermediate standards

In the DXP pathway (Figure 1), pyruvate (PYR) and glyceraldehyde 3-phosphate (GAP) are first condensed by dxs with thiamine to produce 1-deoxy-D-xylulose 5-phosphate (DXP) [4]. DXP is then reduced and isomerized by a single enzyme dxr with NADPH to form 2C-methyl-D-erythritol 4-phosphate (MEP) [4]. MEP reacts with CTP in the presence of ispD to produce 4-diphosphocytidyl-2C-methyl D-erythritol (CDP-ME) [4]. CDP-ME is phosphorylated by an ATP dependent kinase ispE to form 4-diphosphocytidyl-2C-methyl D-erythritol 2-phosphate (CDP-MEP) [4]. CMP is eliminated from CDP-MEP and the molecule is cyclized by ispF to form 2C-methyl-D-erythritol 2,4-diphosphate (MEC) [4]. The ring structure of MEC is opened and reduced by an iron-sulfur cluster containing enzyme ispG by a yet to be fully characterized mechanism to form hydroxylmethylbutenyl diphosphate (HMBPP) [16]. HMBPP is further reduced by another iron-sulfur cluster containing enzyme ispH, to produce a mixture of isopentenyl diphosphate (IPP) and dimethylallyl diphosphate (DMAPP) [17]. Unlike the mevalonate pathway, all the DXP intermediates are phosphorylated and highly acidic. This unique physicochemical property allowed a single step purification of all these metabolites using anionic SPE.

Attempts were initially made to quantify the DXP pathway intermediates by gas chromatography mass spectrometry (GC-MS) after derivatization with trimethylsilyl (TMS). DXP and MEP were readily detected (limit of detection at least 9 pmol per injection). However, all other DXP intermediates were not detectable, likely to be due to the low volatility of the TMS derivatives (File S1). We next explored ultraperformance liquid chromatography mass spectrometry (UPLC-MS) for the quantification of the DXP pathway intermediates.

**Figure 2:**
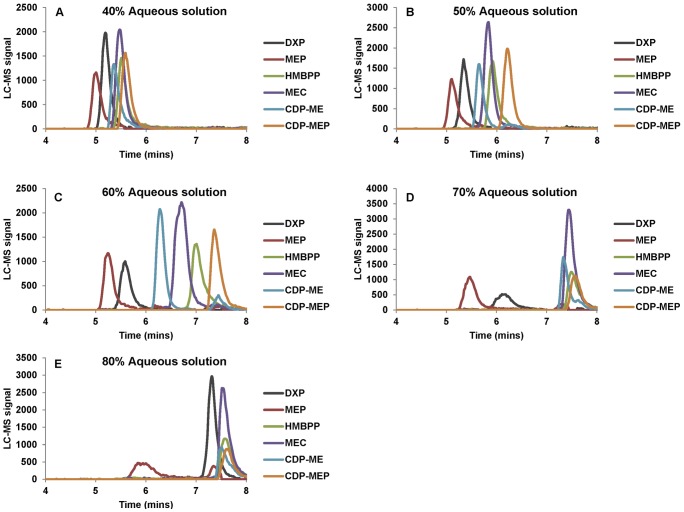
Effects of UPLC gradient on chromatography separation of the DXP pathway intermediates. Different concentrations (0.8, 0.4, 2.6, 2.6, 0.5 and 2 µM respectively) of DXP, MEP, CDP-ME, CDP-MEP, MEC, and HMBPP were prepared in 1 mL acidic extraction solution and purified as described in METHODS AND MATERIALS. Quantification m/z ratio for each compound was extracted from total ion chromatography as Table 1 and traces were overlaid. (A) UPLC gradient described was employed as Table 2 except 40% aqueous solution was used in step 3 and 4; (B) UPLC gradient described was employed as Table 2 except 50% aqueous solution was used in step 3 and 4; (C) UPLC gradient described was employed as Table 2; (D) UPLC gradient described was employed as Table 2 except 70% aqueous solution was used in step 3 and 4; (E) UPLC gradient described was employed as Table 2 except 80% aqueous solution was used in step 3 and 4.

A time-of-fight mass spectrometry was selected because it has the highest scan speed and excellent mass accuracy as compared to other types of mass spectrometry [18]. A range of mass-to-charge ratio (m/z) was scanned in the negative mode and synthetic standards were characterized. All DXP pathway intermediates can be detected and their m/z were unambigously determined (Table 1). Separation of the DXP pathway intermediates was then optimized on a UPLC C18 column (1.7 µm particle size). Ion pairing with tributylamine was found to be an ideal ion-pair reagent allowing excellent resolutions of the various intermediates rapidly (10 mins per sample; Table 2; Figure 2).

**Table 1:** Analytical performance of the SPE UPLC-MS method.

Compound	Retention time (mins)	Quantification ions [M -H]^−^	Linearity		LOQ (µM)	Repeatability (0.1 µM)*	
			Range (µM)	R^2^		Intraday variation (n = 5, CV%)	Interday variation (n = 4, CV%)
DXP	5.6	213.0170±0.03	0.01–2	0.9934	0.01	6.85	7.71
MEP	5.2	215.0330+0.01–0.03	0.01–2	0.9956	0.01	4.69	7.83
CDP-ME	6.2	520.0730±0.03	0.01–0.5	0.9941	0.01	3.90	7.62
CDP-MEP	7.3	600.0390±0.03	0.1–1	0.9902	0.1	10.91	11.63
MEC	6.6	276.9884±0.03	0.02–2	0.9991	0.02	6.20	5.63
HMBPP	7.0	260.9920±0.03	0.01–2	0.9959	0.01	6.54	9.97

* Concentration of CDP-MEP for intra-day/inter-day variation was 0.25 µM.

**Table 2:** Mobile phase gradient used for the separation of DXP intermediates in the UPLC method.

Step	Time (mins)	Aqueous solution*	Methanol
1	0	100%	0
2	1.8	100%	0
3	3.1	60%	40%
4	4.9	60%	40%
5	5.4	10%	90%
6	9.5	10%	90%
7	10	100%	0

* Aqueous solution: 15 mM acetic acid and 10 mM tributylamine.

### Development of solid phase extraction

Solid phase extraction (SPE) has been widely used to isolate analytes in various biological samples [19], [20], [21]. It is rapid and can enrich analytes under mild conditions without the need for solvent evaporation from the extracts [15]. As all the DXP intermediates of interest are phosphorylated (Figure 1), we explored the use of anion exchange SPE [22]. The direct SPE recoveries of all the intermediates were >40% as compared to the initial concentrations of standards used (File S2). It is worthy to note that the LC-NH2 resin used in this study was superior in performance when compared to other similar supports (data not shown) [22].

To examine the linearity, dynamic range and limit of quantification (LOQ) of the SPE UPLC-MS analysis for the DXP pathway intermediates in cell extracts, mixture of DXP, MEP, CDP-ME, CDP-MEP (*in house* synthesized), MEC, and HMBPP were spiked into 10 mL of cell extracts to final concentrations ranging from 2 µM to 0.01 µM. The extracts were then loaded onto an SPE cartridge and eluted in ammonium hydroxide. The results showed that the UPLC-MS signals of all intermediates were linearly correlated with the initial concentrations in cell extracts (R^2^>0.99). In addition, the method demonstrated the LOQ (LOQ≤0.1 µM), intraday variation and interday variations (CV<12%) allowing precise quantification of the DXP pathway intermediates in biological samples (Table 1).

With the developed method, though LOQ of CDP-MEP (0.1 µM) was much higher than that of the other metabolites examined (0.01 µM), it was still considerably more sensitive than reported for the quantification of mevalonate metabolites (a similar class of compounds to DXP metabolites) at LOQ of 4.17 µM [15]. CDP-MEP was found to be co-eluted with many major cellular phospho-compounds, which is likely to result in lower signal due to the phenomenon of ion suppression (File S3). Nonetheless, most these intermediates were readily detected in the genetically modified strains relevant to metabolic engineering of the DXP pathway.

### Analysis of a lycopene producing *E. coli*

**Figure 3:**
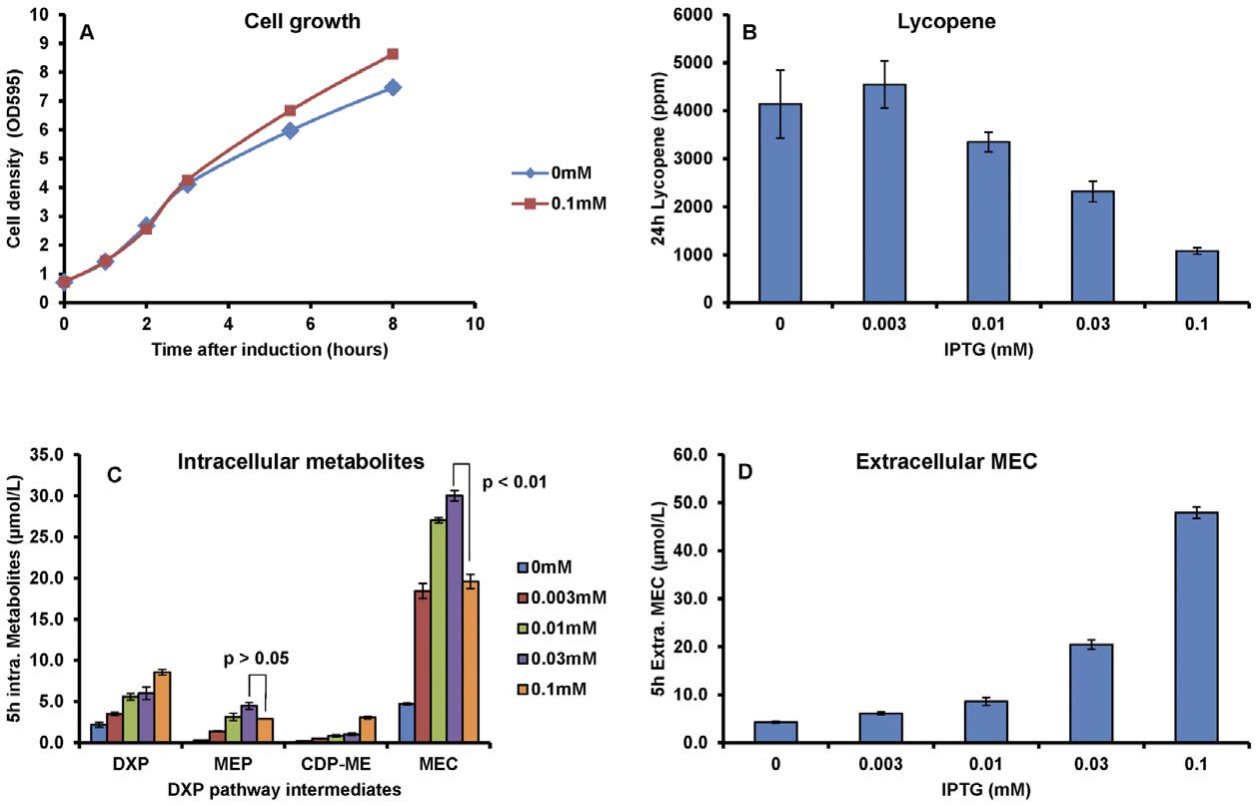
Analysis of *E. coli* expressing DXP pathway genes. E. coli BL21-Gold (DE3) harboring pACLYC and pET-SIDF was induced with IPTG at various concentrations and analyzed by the developed UPLC-MS method. (A) Cell growth in the first 8 hours after IPTG induction; (B) Lycopene at 24 h after induction as a function of IPTG concentration; (C) Intracellular DXP concentration at 5 h after induction as a function of IPTG concentration; (D) Extracellular MEC concentration at 5 h after induction as a function of IPTG concentration; Presented data were average of triplicates (except duplicates for growth curve) and standard errors were drawn on the plot. The Student's t-test was used to calculate the p values.

Lycopene is an antioxidant isoprenoid and the production in *E. coli* is known to be enhanced by overexpression of four enzymes in the DXP pathway (dxs, idi, and ispDF) under non-induced conditions [11]. Consistent with previous studies [3], [8], the strong overexpression of four enzymes (dxs, idi, ispD and ispF) resulted in the inhibition of lycopene production (Figure 3 B). To analyze the profiles of DXP pathway intermediates under this condition, cells were harvested in the exponential phase and the metabolites were quantified. Four intermediates (DXP, MEP, CDP-ME and MEC) were detected in all treatments, and the concentrations increased in parallel to the amounts of inducers, IPTG (Figure 3 C). Interestingly, a significant decrease of intracellular MEC concentration was observed with 0.1 mM IPTG induction (Figure 3 C). Further analysis detected the accumulation of large amount of MEC in the growth medium, greater than the total amount found in the cellular fraction (Figure 3 D). This was an unexpected and intriguing observation as highly charged, phosphorylated compounds, are not known to passively diffuse across intact cell membrane. Similar growth curves were observed with or without 0.1 mM IPTG induction (Figure 3 A), indicative that the viabilities of the cells were not observably different. The high accumulation of MEC in the broth thus could be selectively effluxed from the cells. Preliminary studies showed that the fsr efflux pump may be involved in this process (File S4). It is then not unreasonable to speculate that MEC accumulates to an intracellular threshold concentration (∼30 µmol/L, Figure 3 C) resulting in cellular stress response, as suggested by a recent study of MEC in response to oxidative stress [23]. This may in turn result in the efflux of MEC so as to prevent further intracellular accumulation of this metabolite.

Although the exogenous addition of MEC to levels similar to the amounts effluxed did not affect cell growth and lycopene production (File S5), efflux of MEC itself may have diverted carbon source away from biosynthesis of isoprenoid products resulting in the decrease in isoprenoid biosynthesis. The good inverse correlation between lycopene production and the concentration of extracellular MEC at various IPTG inductions was consistent with this suggestion (Figure 3 B and D).

### Rational engineering of the DXP pathway in *E. coli* for lycopene production

**Figure 4:**
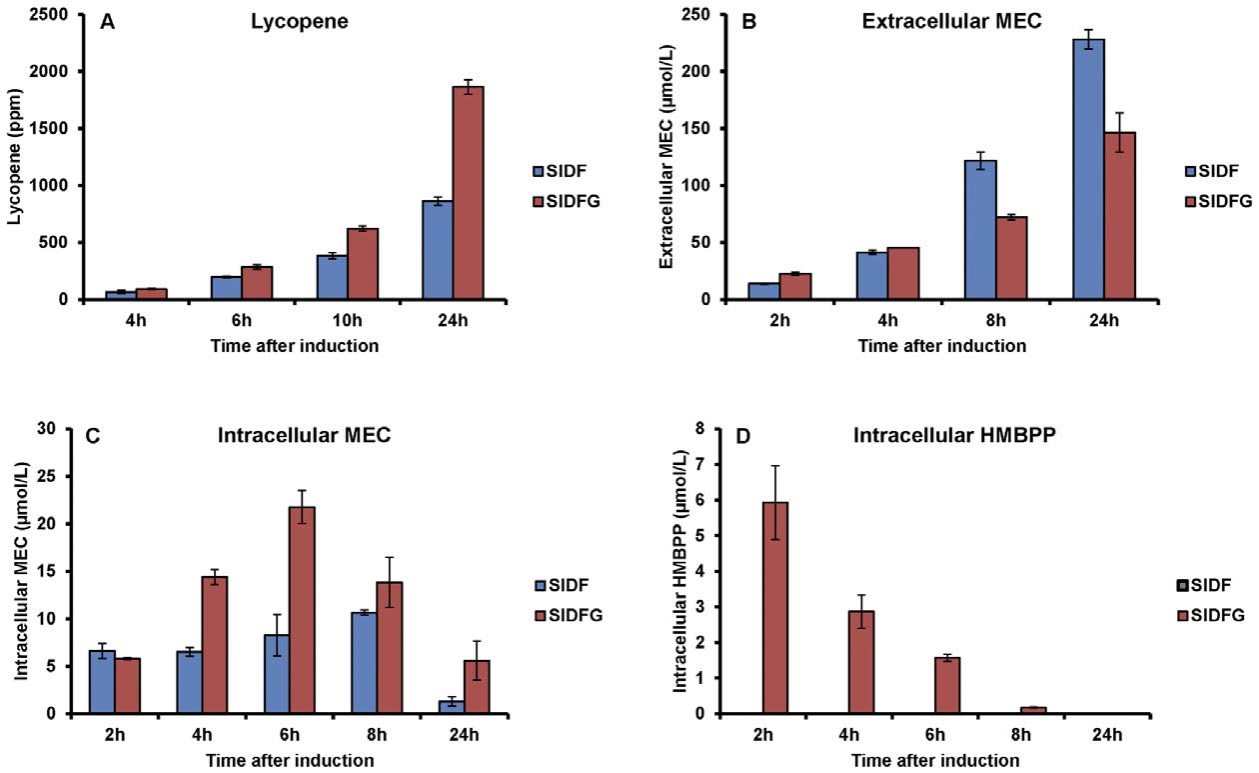
Overexpression of ispG to alleviate efflux of MEC for lycopene production. SIDF: BL21 Gold (DE3) harboring pET-SIDF and pACLYC; SIDFG: BL21 Gold (DE3) harboring pET-SIDFG and pACLYC. (A) Lycopene production as a function of time; (B) Extracellular MEC concentration as a function of time; (C) Intracellular MEC concentration as a function of time; (D) Intracellular HMBPP concentration as a function of time. Presented data were average of triplicates and standard errors were drawn on the plot.

**Figure 5:**
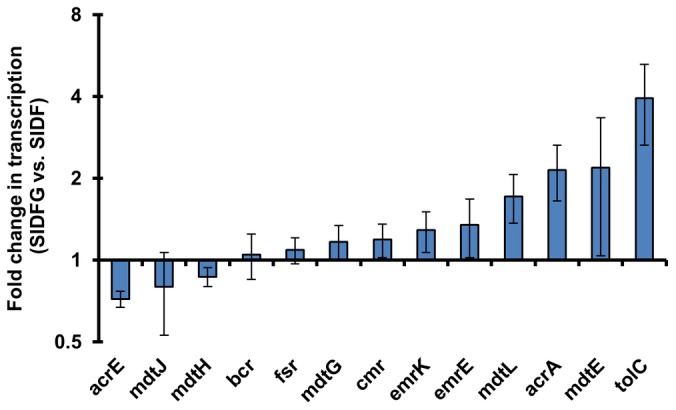
Efflux pumps were not repressed in the strain overexpressing dxs-idi-ispDF-ispG. Transcription fold change of efflux pump encoding genes upon ispG overexpression; bcr, mdtJ, mdtH, mdtA, mdtG, cmr, emrK, emrE, fsr, mdtL and acrA were known genes encoding *E. coli* efflux pumps. Transcriptions of the following efflux pump encoding genes were too low to be accurately quantified in this study: cusC, macA, mdtK, mdtA, mdtM and emrD. They were reported not to be expressed in regular aerobic conditions [24]. Presented data were average of triplicates and standard errors were drawn on the plot.

To test whether lycopene production inhibition was related to MEC efflux, the strain was engineered to co-overexpress ispG (with dxs-idi-ispDF), the enzyme utilizing MEC (Figure 1). In the strong induction condition (0.1 mM IPTG), where lycopene production was severely inhibited and MEC efflux was high (Figure 3 B and D), co-overexpression of ispG indeed concurrently increased lycopene production (Figure 4 A) and reduced efflux of MEC (Figure 4 B). The concentration of intracellular MEC was found to be increased with ispG overexpression, a result supporting the proposal of an intimate relationship between ispG and intracellular MEC. HMBPP (a product of ispG, Figure 1) was also readily detected inside the cells overexpressing ispG (HMBPP was not detectable in the parental strain expressing dxs-idi-ispDF, Figure 4 D), consistent with the above proposal that with higher ispG more MEC was consumed for isoprenoid biosynthesis. In order to gain an insight into the profiles of efflux pumps in these strains, all the 20 known efflux pump operons in *E. coli* [24] were analyzed at the transcription level. The expression levels of these pumps were not regulated by ispG overexpression (Figure 5). Taken together, overexpression of the metabolic enzyme ispG reduced the amount of MEC efflux possibly by shuttling it directly to the downstream products, e.g. lycopene.

### Analysis of bacteria other than *E. coli*

**Figure 6:**
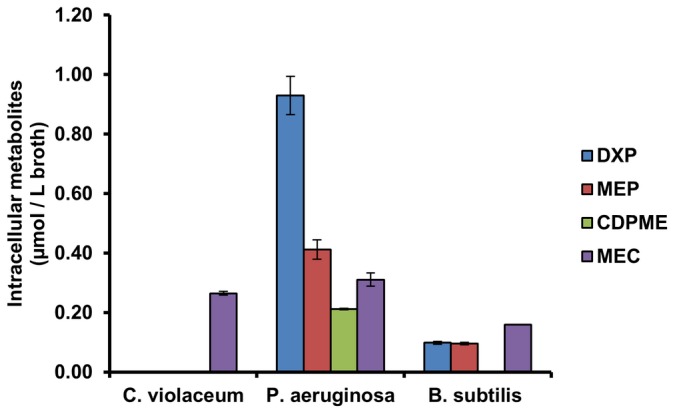
Analysis of bacteria other than *E. coli*. Intracellular DXP pathway intermediates at middle exponential growth phase. Presented data were average of triplicates and standard errors were drawn on the plot. *C. violaceum*: *Chromobacterium violaceum; P. aeruginosa : Pseudomonas aeruginosa; B. subtilis : Bacillus subtilis*.

To demonstrate the broader utility of the method developed here, measurements of the DXP metabolites were conducted in other bacteria. Besides *E. coli*, other bacteria have also been used for the production of isoprenoids [25]. The DXP metabolites of two gram negative strains (*Chromobacterium violaceum* [26] and *Pseudomonas aeruginosa* [27]) and one gram positive strain (*Bacillus subtilis* [25]) were characterized. A gram positive strain (*Staphylococcus aureus* [28]) that utilize the MVA pathway but not the DXP pathway was also included as a negative control. Analysis of the cells harvested in the exponential growth phase (OD595 = 2–3) indeed showed that intracellular DXP pathway intermediates can be detected in all strains except *Staphylococcus aureus* (Figure 6). The four bacteria used in this study were all wild types, where the DXP pathway genes were not genetically overexpressed. The level of the DXP pathway intermediates in these strains was expectedly lower than that in engineered *E. coli*, yet the developed method here has the sensitivity to measure these intermediates. None of the DXP pathway intermediates were detected in the broth supernatant of these strains (data not shown), suggesting that no DXP intermediates were effluxed from these cells under the conditions.

## Discussion

The engineered DXP pathway has been demonstrated to be a powerful synthetic platform for microbial production of valuable isoprenoids. However, current strategies in manipulation of this pathway are still mostly combinatorial in nature, which are largely due to the lack of methods for conveniently monitoring changes of the pathway in metabolite level. Although a LC-MS/MS method was recently reported for measuring some of the DXP metabolites [29], a more sensitive and convenient method was still desirable because the LC-MS/MS method had moderate sensitivities (∼5 µM for most metabolites) and involved liquid-liquid extraction in sample preparation. In this study, the use of UPLC-MS, for the first time, has enabled the detection of all the DXP metabolites. Coupled to an anion exchange solid phase extraction protocol, the method was able to quantify all the DXP metabolites in biological samples with good sensitivity (LOQ≤0.1 µM) and reliability (intraday variation CV≤12%). Sample preparation in our method was also rapid (5 mins) and easy (drying free), demonstrating the superiority of the SPE UPLC-MS method developed in this study. In the analyzed biological samples, all the DXP metabolites except CDP-MEP were detected (Figure 3 C and Figure 4 D). A strain overexpressing dxs and ispE (the enzyme to produce CDP-MEP) was constructed for the purpose of demonstration in quantifying the amount of endogenous CDP-MEP. However, the metabolite was still undetectable. It is likely that ispE and ispF formed protein complex in *E. coli* [30] and consumed most CDP-MEP once it was produced.

**Figure 7:**
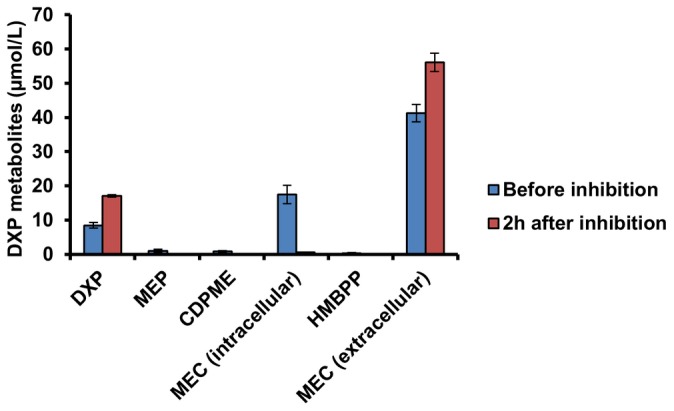
The effects of fosmidomycin inhibition of dxr on DXP metabolism. Twenty five microgram per milliliter fosmidomycin was used to inhibit MEC biosynthesis at 4 h after induction, and the metabolites of BL21 Gold (DE3) harboring pET-SIDF and pAC-LYC were analyzed before and 2 h after inhibition; Presented data were average of triplicates and standard errors were drawn on the plot.

Beyond the method development, this study has also significantly contributed to the body of knowledge of the DXP metabolism in isoprenoid production. In the engineered lycopene producing *E. coli*, MEC was found to be actively effluxed, serving as a previously unrecognized branch in the DXP pathway. Kinetics of this competing branch pathway was estimated by inhibiting MEC synthesis with fosmidomycin and measuring the amounts of accumulated intracellular MEC. It was found that a portion of the accumulated MEC was actually effluxed within 2 hours instead of being utilized by the cells (Figure 7), indicative that MEC efflux served as a competing pathway diverting carbon source away from the isoprenoid biosynthesis (consistent with the inverse correlation between Figure 3 B and D). From a theoretical consideration, the amounts of MEC accumulated in the media at 24 h after induction was ∼220 µmol/L (Figure 4 B), which was calculated to be equivalent to 4500 ppm lycopene and significantly greater than the amount of lycopene produced (1000 ppm, Figure 4 A). As a rational strategy, ispG was then overexpressed and the efflux of MEC was successfully reduced, resulting in the increase of the downstream intermediates (HMBPP etc.) and the isoprenoid product (lycopene). These findings have demonstrated the usefulness of the developed SPE UPLC-MS method. Future studies to effectively divert the amount of MEC effluxed into isoprenoid biosynthesis will invariably be valuable to the engineering of microbes for the overproduction of isoprenoids.

### Conclusion

In this study, an anion exchange solid phase extraction was developed to efficiently and selectively purify all the DXP pathway intermediates from biological samples simultaneously. UPLC-MS (TOF) was then optimized to reliably quantify the purified intermediates with good sensitivity. The method allows the direct monitoring of DXP metabolites in cells for the production of isoprenoids, an invaluable tool for rational engineering of the pathway which is currently not available.

## Materials and Methods

### Bacteria strains and plasmids

*E. coli* BL21-Gold (DE3) (Stratagen) [*E. coli* B F^−^ ompT hsdS (r_B_^−^ m_B_^−^) dcm^+^ Tet^r^ gal λ(DE3) endA Hte] was used for lycopene production with pACLYC [31]. pET-SIDF (named as p20T7MEP in [3]) was transformed into BL21-Gold (DE3) harboring pACLYC. IspG was amplified by polymerase chain reaction with the extracted *E. coli* genomic DNA. Primers XhoI-RBS-ispG F and ispG-BamHI R were used (primers used in this study are summarized in File S6). The purified PCR product was ligated into pET-SIDF which contained XhoI site and BamHI site. The resulting plasmid was termed as pET-SIDFG, which was subsequently transformed into BL21-Gold (DE3) with pACLYC.

*E. coli* DH5α (Invitrogen) [F- φ80lacZΔM15 Δ(lacZYA-argF)U169 recA1 endA1 hsdR17(rk^−^, mk^+^) phoA supE44 thi-1 gyrA96 relA1 λ^−^] was used for genomic DNA extraction. *E. coli* XL10-Gold (Stratagene) [Tet^r^ Δ(mcrA)183 Δ(mcrCB-hsdSMR-mrr)173 endA1 supE44 thi-1 recA1 gyrA96 relA1 lac Hte [F′ proAB lacI^q^ZΔM15 Tn10 (Tet^r^) Tn5 (Kan^r^) Amy]] with pACLYC was used for production of recombinant ispE enzymes. IspE was amplified by polymerase chain reaction with the extracted *E. coli* genomic DNA. Primers SacI-ispE F and ispE-XhoI R were used (File S6). The purified PCR product was ligated into a modified pBAD-B (Invitrogen) which contained six histidine tag, SacI site and XhoI site. The resulting plasmid was termed as pBAD-ispE, which was subsequently transformed into *E. coli* XL10-Gold with pACLYC.

Wild type *Bacillus subtilis* (BGSC 1A1) was requested from BGSC. Wild type *Chromobacterium violaceum, Pseudomonas aeruginosa* and *Staphylococcus aureus* were gifts from Prof. Yew Wen Shan (Department of Biochemistry, NUS).

### Synthesis of CDP-MEP

Standards of all the DXP pathway intermediates are commercially available (Echelon) except CDP-MEP which was synthesized *in house* as described in this session. Recombinant ispE was purified from *E. coli* XL10-Gold containing pBAD-ispE and pACLYC. A colony was picked from agar plate, inoculated into 2xPY medium (20 g/L peptone, 10 g/L yeast extract and 10 g/L NaCl, pH = 7) containing 34 µg/mL chloramphenicol and 100 µg/mL ampicillin, and incubated overnight. Five hundred microliter aliquots of cell culture grown overnight were inoculated into 25 mL 2xPY medium in 250 mL shake flask. Cells were grown at 37°C/300 rpm until OD595 reached between 0.5∼1.0. The cells were then induced with 10 mM L-arabinose and grown at 20°C for 24 hours. The cell pellets were collected and resuspended in 1 mL B-PER II reagent (Pierce) and homogenized on Mini-Beadbeater-16 (Biospec) with 200 µL glass beads for 1 min. The cell lysates were centrifuged for 10 min at 4°C and the supernatant was diluted with 20 mL NPI-10 (50 mM Na_2_HPO_4_, 300 mM NaCl and 10 mM imidazole, pH = 8) and incubated with 200 mg Ni-NTA micro beads (USB) for 1 h at room temperature. The beads were washed with 40 mL NPI-10 and eluted with 300 µL NPI-250 (50 mM Na_2_HPO_4_, 300 mM NaCl and 250 mM imidazole, pH = 8).

In a 50 µL reaction, 1.25 mM CDP-ME was converted into CDP-MEP with 10.5 µg recombinant ispE in the presence of 40 mM Tris (pH = 6), 2.5 mM MgCl_2_, 50 mM β-mercaptoethanol and 10 mM ATP. The reaction was incubated at 37°C for 15 h and terminated with 450 µL acidic extraction solution (acetonitrile/methanol/water 40∶40∶20+0.1 M formic acid). CDP-MEP was confirmed by mass spectrometry and the concentration was estimated by measuring consumed CDP-ME in the reaction.

### *E. coli* growth and induction of protein expression

A colony was picked from agar plate, inoculated into 2xPY medium containing 34 µg/mL chloramphenicol and 100 µg/mL ampicillin, and incubated overnight. Fifty microliter aliquots of cell culture grown overnight were inoculated into 5 mL 2xPY medium in 50 mL Falcon tube. Cells were grown at 37°C/300 rpm until OD595 reached between 0.5∼1.0. The cells were then induced with IPTG at the indicated concentrations and grown at 28°C for the given time periods prior to collection for metabolic extraction or lycopene assays.

### Extraction and quantification of lycopene

Fifty microliters of cell suspension was sampled at 24 h after induction, and the OD595 was recorded. The cells were centrifuged for 2 min and resuspended in 200 µL of acetone. Resuspended cells were vortexed for 10 min and centrifuged at 3,000 g for 2 min. One hundred microliters of supernatant was mixed with equal volume of ethanol and transferred to 96 well optical bottom plate (NUNC). Lycopene content was determined by interpolating from a standard dilution of lycopene (Sigma) based on absorbance at 472 nm (Spectra Max 190, Molecular Devices).

### Solid phase extraction of the DXP pathway intermediates in biological samples

Cell suspension equivalent to 6 mL OD595 = 1.0 cells were withdrawn. The cells were centrifuged for 5 min. The cell pellets were resuspended in 10 mL acidic extraction solution (acetonitrile/methanol/water 40∶40∶20 +0.1 M formic acid) [32] for intracellular metabolite extraction. One hundred microliter supernatant was diluted with 100 µL ddH2O and 800 µL modified acidic extraction solution (acetonitrile/methanol 50∶50 +0.125 M formic acid) for extracellular metabolite extraction. The resuspended cell pellets in acidic extraction solution was incubated at −20°C for 60 min with periodic shaking. Following centrifugation, the supernatant was purified through a LC-NH2 resin (Sigma). Diluted broth supernatant was also purified by LC-NH2 resin in the same way. The cartridge was eluted with 400 µL 1% NH_4_OH solution after centrifugation at 3,000 g for 2 min and pH of eluate was subsequently adjusted to acidic (pH = 5) by 3 µL acetic acid.

### UPLC – MS quantification of the DXP pathway intermediates

A LC-MS/MS method was developed for phosphorylated sugars with HPLC MS/MS [14]. The method was modified for DXP intermediates with UPLC (Waters ACQUITY UPLC) – (TOF)MS (Bruker micrOTOF II) platform. In brief, aqueous solution containing 15 mM acetic acid and 10 mM tributylamine and methanol were used as mobile phase with a UPLC C18 column (Waters CSH C18 1.7 µm 2.1×50 mm). The elution was done at 0.15 mL/min (Table 2). Electrospray ionization was used and mass spectrometry was operated to scan 50–800 m/z in negative mode with −500 V end plate voltage and 4500 V capillary voltage. Nebulizer gas was provided in 1 bar, drying gas temperature was 9 mL/min, and dry gas temperature was 200°C. Sample injection volume was 5 µL.

A range of m/z typically with 0.06 m/z width (the average m/z distribution width with the used MS instrument) was extracted from the acquired data (50–800 m/z) for each intermediate. The range of m/z was determined with 50 µM standards of each intermediate prepared individually in water except CDP-MEP which was in elution solution of SPE. At the assay condition, all the intermediates were detected in the form [M-H]^−^. Retention time was subsequently determined for each intermediate with the same 50 µM standards and the set m/z extraction range. The peak area was calculated with the software provided by the manufacturer. Based on the peak area, concentrations of the DXP pathway intermediates were determined from calibration curves constructed by using synthetic standards prepared in biological matrix (cell extracts or broth supernatant).

Linearity of the assays was determined with cell extracts containing DXP, MEP, CDP-ME, CDP-MEP, MEC and HMBPP in concentration of 2 µM, 1 µM, 0.5 µM, 0.25 µM, 0.1 µM, 0.05 µM, 0.02 µM and 0.01 µM. Intraday/interday variations were determined with indicated number of replicates and sample concentration as Table 1.

### Transcription quantification

Cell suspension equivalent to 0.1 mL OD595 = 1.0 cells was sampled at 4 h after induction. Gene transcription was quantified as described in [11], where cysG, idnT and hcaT were used as reference genes. Primers used were summarized in File S6.

### Fosmidomycin inhibition of MEC biosynthesis

Fosmidomycin was added into the cell in the final concentration of 25 µg/mL at 4 h after the IPTG induction. Intracellular and extracellular metabolites were analyzed at 2 h after the fosmidomycin inhibition.

### Growth of bacteria other than *E. coli*

Glycerol stocks of *Bacillus subtilis*, *Chromobacterium violaceum, Pseudomonas aeruginosa* and *Staphylococcus aureus* were directly inoculated into 2xPY and incubated at 37°C overnight. Cell pellets of cells grown overnight were resuspended in 10 mL 2xPY (OD595 = 0.2) in 50 mL Falcon tube. Cells were grown at 37°C/300 rpm until OD595 reached between 2 and 3, when cells were collected for metabolite quantifications.

## Supporting Information

File S1 **GC-MS analysis of the DXP pathway intermediates.**
(DOC)

File S2 **Recovery of the solid phase extraction of the DXP pathway intermediates.**
(PPT)

File S3 **ATP co-eluted with CDP-MEP and affected its detection with the SPE UPLC-MS method.**
(PPT)

File S4 **Investigation of biochemical mechanism of the MEC efflux in engineered** ***E. coli*** **.**
(DOC)

File S5 **Investigation of biochemical impact of extracellular MEC.**
(DOC)

File S6 **Primers used in this study.**
(DOC)
