# Supplementary material for: Metabolite Profiling Identified Methylerythritol Cyclodiphosphate Efflux as a Limiting Step in Microbial Isoprenoid Production
Source: PLoS One. 2012 Nov 2;7(11):e47513. doi: 10.1371/journal.pone.0047513 (PMC3487848; doi:10.1371/journal.pone.0047513)
Supplement: File S1 — GC-MS analysis of the DXP pathway intermediates. (DOC) [file pone.0047513.s001.doc]

# Supplementary file 1 GC-MS analysis of the DXP pathway intermediates

Gas chromatography mass spectrometry (GC-MS) has been widely used in the analysis of intracellular metabolites with trimethylsilyl TMS derivatization . The standard protocol as reported in was employed in this study. Five microliter 2.5 mM DXP, MEP, CDP-ME, MEC and HMBPP was individually dried and incubated with 25 µL MOX reagent (Thermo Scientific) at 37 °C for 1 hour. Twenty five microliter MSTFA + 1% TMCS (Thermo Scientific) was subsequently added and incubated at 37 °C for 1 hour. Samples were centrifuged at 16,000g for 1 min and 1 µL sample containing 250 pmol metabolite was injected to GC-MS in splitless mode at 270 °C. Agilent 7890 GC equipped with HP-5ms GC column and Agilent MS were used at flow rate 1 mL / min. Oven temperature was held at 100 °C for 2mins, elevated to 300 °C at 40 °C / min and held at 300 °C for 3 mins. MS source and quadrupole temperature were 230 °C and 150 °C respectively. MS data was acquired in scan mode from 100 to 500 m/z and data analysis was done with AMDIS Chromatogram (NIST). DXP was detected at 6.70 mins with signature m/z 290, 315 and 357; MEP was detected at 6.84 mins with signature m/z 299, 315 and 387. CDP-ME, MEC and HMBPP were not detected and thus structurally more complicated CDP-MEP was not tested. DXP and MEP were then serial diluted and analyzed by GC-MS. The limit of detection was at least 9 pmol per injection for DXP and MEP, and linearity was satisfactory (Supplementary figure S1). Intraday variation of DXP and MEP were 6.5% and 6.8% respectively at 28 pmol per injection. Because GC-MS is not suitable for most of the DXP pathway intermediates, further characterization of this this method was not carried out.


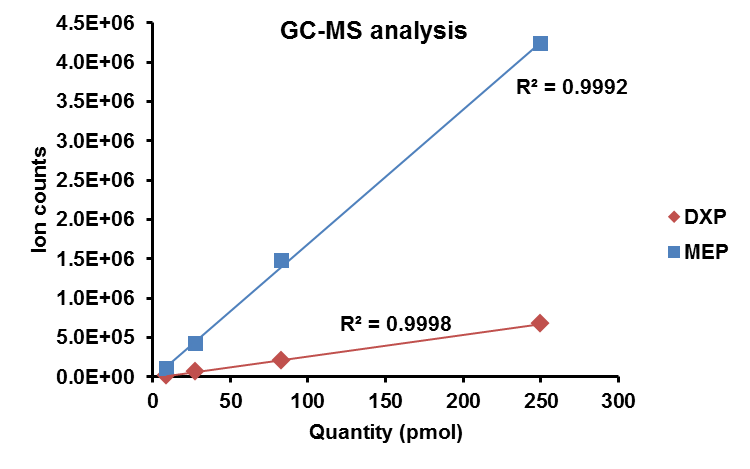


Supplementary figure S1 Characterization of GC-MS analysis of DXP and MEP standards

## References

1. Hiller K, Metallo CM, Kelleher JK, Stephanopoulos G (2010) Nontargeted Elucidation of Metabolic Pathways Using Stable-Isotope Tracers and Mass Spectrometry. Anal Chem.
