# Supplementary material for: Metabolite Profiling Identified Methylerythritol Cyclodiphosphate Efflux as a Limiting Step in Microbial Isoprenoid Production
Source: PLoS One. 2012 Nov 2;7(11):e47513. doi: 10.1371/journal.pone.0047513 (PMC3487848; doi:10.1371/journal.pone.0047513)
Supplement: File S2 — Recovery of the solid phase extraction of the DXP pathway intermediates. (PPT) [file pone.0047513.s002.ppt]

## Slide 1
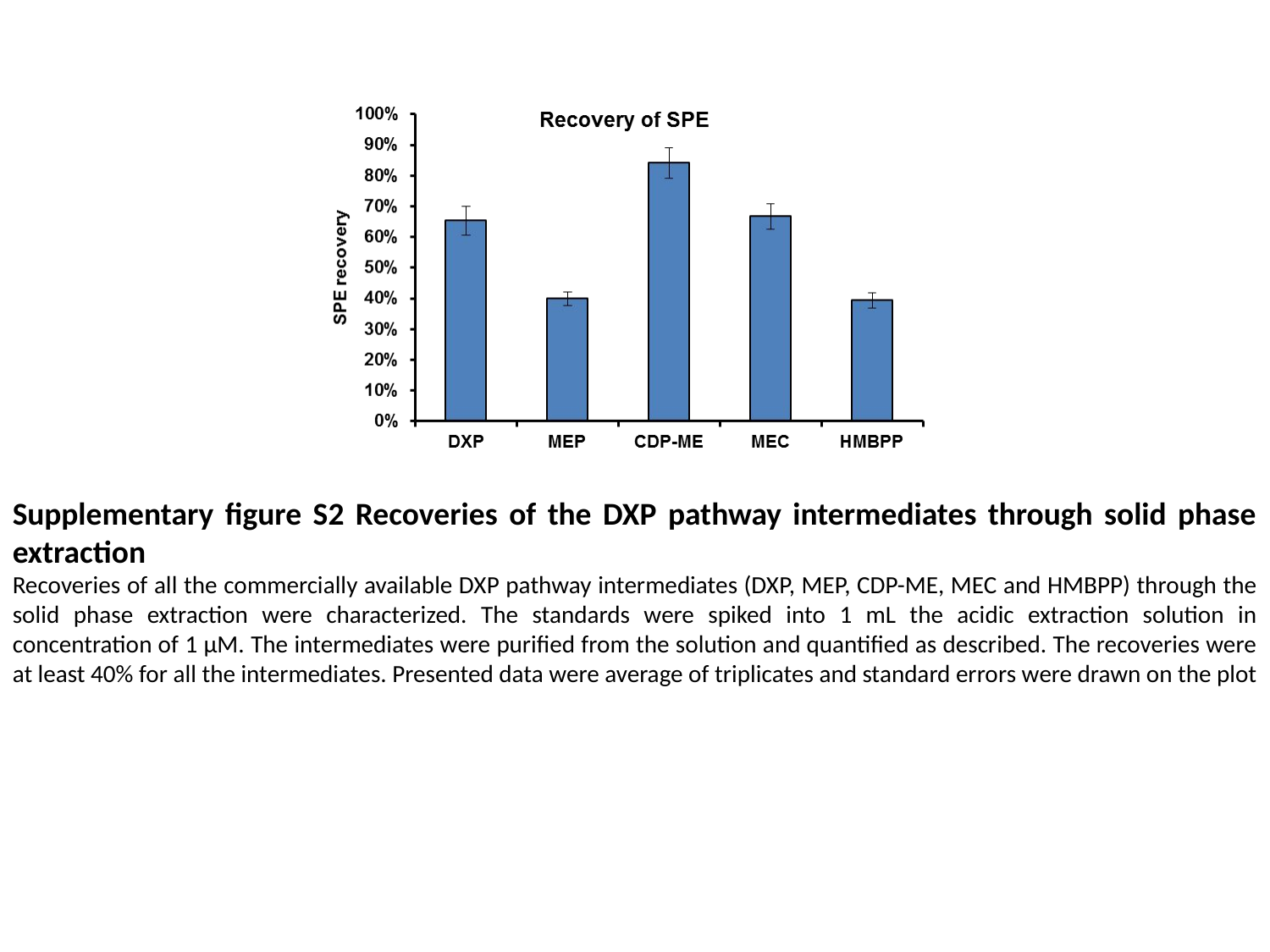

Supplementary figure S2 Recoveries of the DXP pathway intermediates through solid phase extraction
Recoveries of all the commercially available DXP pathway intermediates (DXP, MEP, CDP-ME, MEC and HMBPP) through the solid phase extraction were characterized. The standards were spiked into 1 mL the acidic extraction solution in concentration of 1 µM. The intermediates were purified from the solution and quantified as described. The recoveries were at least 40% for all the intermediates. Presented data were average of triplicates and standard errors were drawn on the plot
