# Supplementary material for: Metabolite Profiling Identified Methylerythritol Cyclodiphosphate Efflux as a Limiting Step in Microbial Isoprenoid Production
Source: PLoS One. 2012 Nov 2;7(11):e47513. doi: 10.1371/journal.pone.0047513 (PMC3487848; doi:10.1371/journal.pone.0047513)
Supplement: File S3 — ATP co-eluted with CDP-MEP and affected its detection with the SPE UPLC-MS method. (PPT) [file pone.0047513.s003.ppt]

## Slide 1
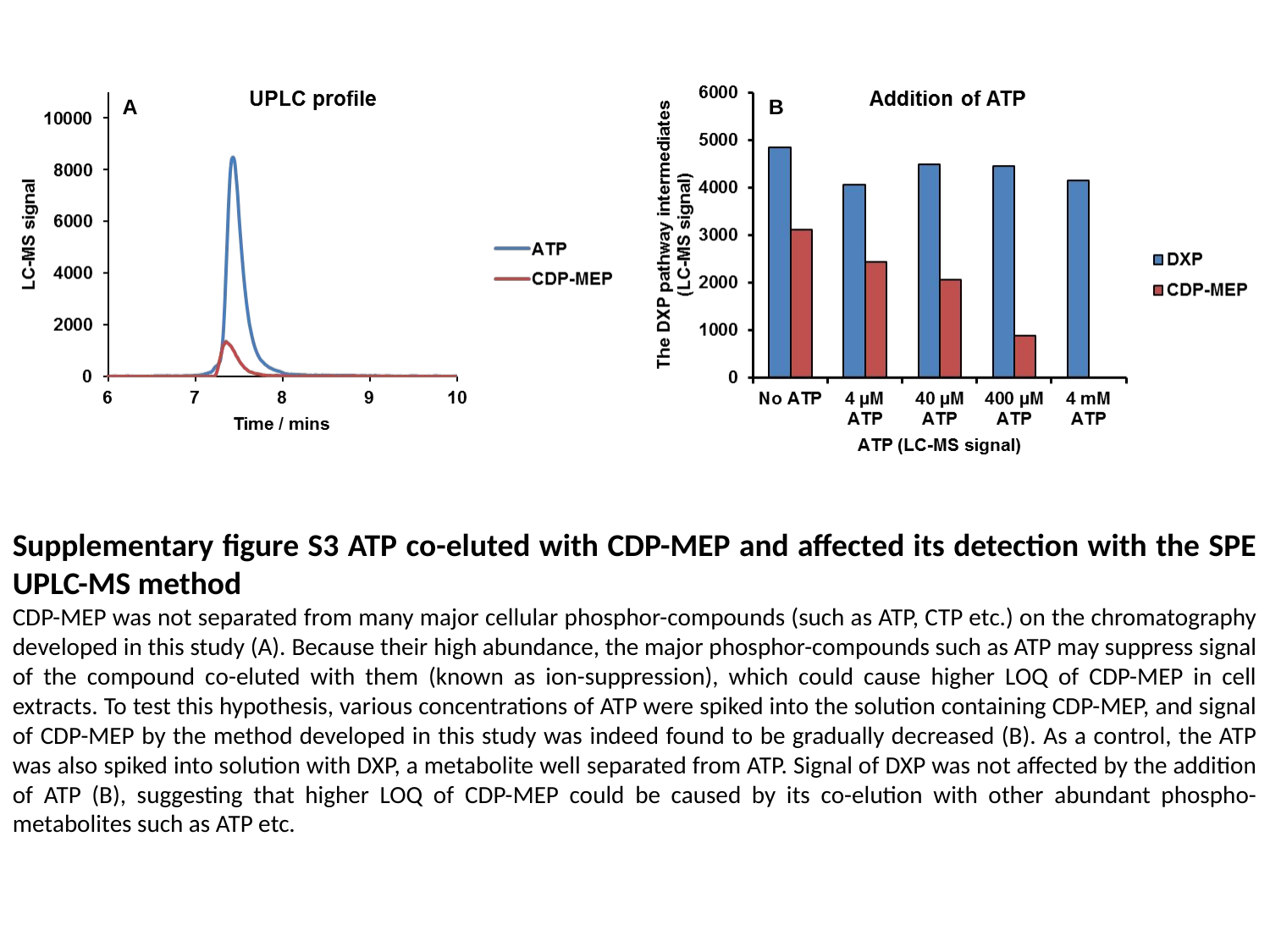

A
B
Supplementary figure S3 ATP co-eluted with CDP-MEP and affected its detection with the SPE UPLC-MS method
CDP-MEP was not separated from many major cellular phosphor-compounds (such as ATP, CTP etc.) on the chromatography developed in this study (A). Because their high abundance, the major phosphor-compounds such as ATP may suppress signal of the compound co-eluted with them (known as ion-suppression), which could cause higher LOQ of CDP-MEP in cell extracts. To test this hypothesis, various concentrations of ATP were spiked into the solution containing CDP-MEP, and signal of CDP-MEP by the method developed in this study was indeed found to be gradually decreased (B). As a control, the ATP was also spiked into solution with DXP, a metabolite well separated from ATP. Signal of DXP was not affected by the addition of ATP (B), suggesting that higher LOQ of CDP-MEP could be caused by its co-elution with other abundant phospho-metabolites such as ATP etc.
