# Supplementary material for: Metabolite Profiling Identified Methylerythritol Cyclodiphosphate Efflux as a Limiting Step in Microbial Isoprenoid Production
Source: PLoS One. 2012 Nov 2;7(11):e47513. doi: 10.1371/journal.pone.0047513 (PMC3487848; doi:10.1371/journal.pone.0047513)
Supplement: File S4 — Investigation of biochemical mechanism of the MEC efflux in engineered E. coli . (DOC) [file pone.0047513.s004.doc]

# Supplementary file S4 Investigation of biochemical mechanism of the MEC efflux in engineered *E. coli*

It was speculated based on the cell growth (Figure 3 A) and the chemical property of MEC that the metabolite may likely be actively effluxed. To test this hypothesis, we took advantage of a recent created collection of single-gene deletion *E. coli* in Coli Genetic Stock Center (Yale University), where all the nonessential genes were individually knocked out in *E. coli* BW25113 strain. Three mutants (∆fsr, ∆acrB and ∆cusC) were first characterized and validated to be deficient in the expression of these genes. The genes (dxs-idi-ispDF) were then maximally overexpressed in these 3 mutants and the parental wild type strain. The extracellular MEC concentration of the mutant ∆fsr was found to be significantly lower as compared to that of the parental strain (~ 20% of the level of the parental strain, Supplementary figure S5). The data was consistent with the hypothesis that MEC was actively exported by this efflux pump and further studies will be required to identify the contributions of the other pumps not examined in this preliminary study. In addition, the data here also demonstrated the generality of the MEC efflux phenomenon, as BW 25113 is an *E. coli* K-12 derivative strain, distinct from BL21 (Figure 3).


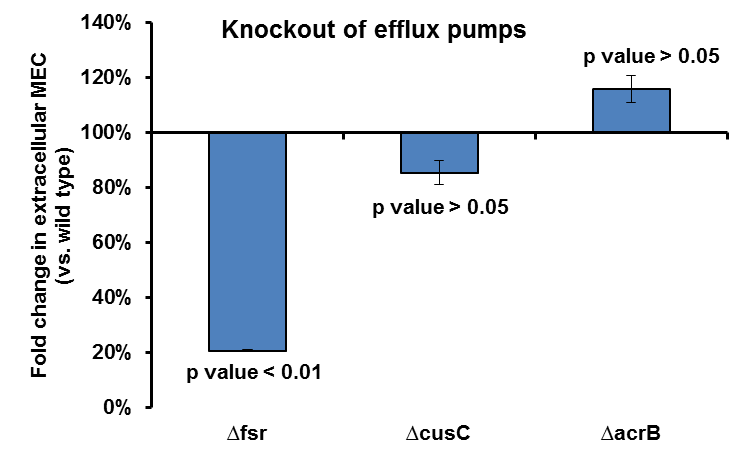


Supplementary figure S5 Addition of MEC does not affect production of lycopene

Extracellular MEC concentration of the mutants (∆fsr, ∆acrB and ∆cusC) at 24h after induction was compared to that of their parental strain (77 ± 1.4 µM). Extracellular MEC concentrations were normalized to cell density. Presented data were average of triplicates and standard errors were drawn on the plot. Student’s t-test were used to calculate the p values, and only the changes of the ∆fsr strain was significant.
