# Supplementary material for: Metabolite Profiling Identified Methylerythritol Cyclodiphosphate Efflux as a Limiting Step in Microbial Isoprenoid Production
Source: PLoS One. 2012 Nov 2;7(11):e47513. doi: 10.1371/journal.pone.0047513 (PMC3487848; doi:10.1371/journal.pone.0047513)
Supplement: File S5 — Investigation of biochemical impact of extracellular MEC. (DOC) [file pone.0047513.s005.doc]

# Supplementary file S5 Investigation of biochemical impact of extracellular MEC

To further understand the impact of accumulated MEC in the media on the cell growth and lycopene production, synthetic MEC was spiked into the media of the *E. coli* BL21 overexpressing dxs-idi-ispDF at the uninduced condition. Lycopene production and growth of the cells were not significantly affected by the addition of MEC (Supplementary figure S5 A and B), suggesting that extracellular MEC does not regulate the production of lycopene. The concentrations of extracellular MEC at 14h after the MEC spiking were found to be similar to the initial spiked concentrations (Supplementary figure S5 B), indicative that extracellular MEC was not consumed by the uninduced cells. Once the cells overexpressing dxs-idi-ispDF was induced by IPTG, significant amount of extracellular MEC was produced (Figure 3 D), which would be mixed with the spiked-in MEC and cannot be distinguished by the current method. Therefore, impact of extracellular MEC on the cells was only investigated in the uninduced conditions. In the future studies, the cells could be grown on 13C carbon source, and the endogenous MEC (13C MEC) would be easily distinguished from the spiked-in 12C MEC, which will enable examination of impact of extracellular MEC in the induced cells.


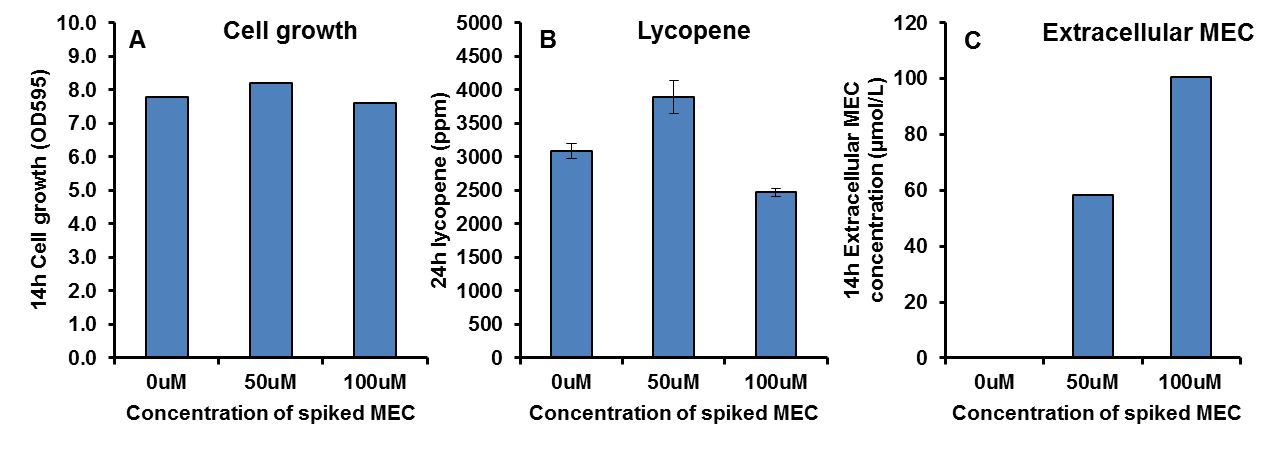


Supplementary figure S5 Addition of MEC does not affect production of lycopene

Different concentrations of MEC was spiked into BL21 harboring pET-SIDF and pAC-LYC in early exponential growth phase (OD595=0.5~1.0). Concentrations of the spiked MEC in the media were 50 and 100 µM, which are in the range of extracellular MEC the cells could produce upon IPTG induction (Error: Reference source not found D). The lycopene production and the extracellular MEC after cell growth were monitored. (A) Cell growth at 14h after the MEC spiking as a function of the spiked MEC concentration; (B) Lycopene production at 24h after the MEC spiking as a function of the spiked MEC concentration; (C) The extracellular MEC concentration at 14h after MEC spiking
