# Supplementary material for: Metabolite Profiling Identified Methylerythritol Cyclodiphosphate Efflux as a Limiting Step in Microbial Isoprenoid Production
Source: PLoS One. 2012 Nov 2;7(11):e47513. doi: 10.1371/journal.pone.0047513 (PMC3487848; doi:10.1371/journal.pone.0047513)
Supplement: File S6 — Primers used in this study. (DOC) [file pone.0047513.s006.doc]

Supplementary table S1 Primers used in this study

| Primer | Sequence |
| --- | --- |
| XhoI-RBS-ispG F | GCCTCGAGACCATTCATCAGATAAGCAAACTAAGGACTTTCAAATGCATAACCAGGCTCCAA |
| ispG-BamHI R | GCGGATCCTTATTTTTCAACCTGCTGAACG |
| SacI-ispE F | GCTTAGAGCTCCGGACACAGTGGCCCTC |
| ispE-XhoI R | GTAACCTCGAGTTAAAGCATGGCTCTGTGC |
| acrD 1252F  acrD 1413R  acrE 903F  acrE 1054R  bcr 783F  bcr 882R  cmr 1020F  cmr 1161R  cusC 1086F  cusC 1269R  emrD 27F  emrD 169R  emrE 188F  emrE 332R  emrK 872F  emrK 1036R  emrR 170F  emrR 244R  fsr 2F  fsr 104R  macA 544F  macA 705R  mdtA 626F  mdtA 748R  mdtE 439F  mdtE 606R  mdtG 287F  mdtG 399R  mdtH 674F  mdtH 767R  mdtJ 21F  mdtJ 157R  mdtK 1207F  mdtK 1321R  mdtL 438F  mdtL 630R  mdtM 1062F  mdtM 1145R | CGTATTATGAGTGAGGAAGGA  AGAGAACTGGCGATAGATG  ACCTGACGCCATTCTTAT  GTCCTTCACTAATCAACCATT  TCTGTTCGTGATGACCAT  CGCTGCCATAATAAATTGTATC  TGCCAGCGATATGAGTAA  GTTGACAAGGTTGAAGAGAT  GGTGGTGAATTATGAACAGAA  CAGCACTTCCAGATAACTTAC  GTTATTGATGTTGGTATTACTC  CGTAAGTCAGCAGATAAG  GGTCAGGAGTCGGTATTG  TAATGTGGTGTGCTTCGT  ATGCGTTCTCCTTATTACCT  CAATGTCTTCGTTCTTCGTAT  TGTTTATGGCGTTGATTA  CAAGAGCACAACTTAATTC  TGGCAATGAGTGAACAAC  AGATGTGAGAGGCTGATAG  GATTACACTCGCATCGTT  GATTACATCCGCTTCAGAA  AGCAGGTTGATGTTGGTA  GCACTACGGTAGCGATAT  AATGAAGCAGAAGCCAATG  TACGGTAACCAGCGAATC  TCGTGATGGTGTTGATGG  TGTGGCGATAAGAGCATTA  TACAAGTGATGCTGATGCT  GACAGACACGCTTCAATG  ATTAGGTCTGGCTATTGCTA  TAACGGCGAAAGAGAGAA  GGCTATATTCTGGCACTGA  GCATACGCAACATCATCAT  CGGACATCTGATTATGCTTAA  GATAACAACACGGCTGAG  GATGGTGATGTCGGTCTC  ACAACGGCTAACAGATGAA |
| acrA 1119F  acrA 1194R  acrB 3069F  acrB 3148R | AAAGCACAAGAAGTTACC  TTAAGACTTGGACTGTTCA  GTATTCTTTGTGGTGGTT  AATGATGATCGACAGTATG |
| tolC 1049F  tolC 1190R | GTTCCTCCTTCAACAACATTA  GCATCCAACACATCAACA |
| cysG 50F | TTGTCGGCGGTGGTGATGTC |
| cysG 154R | ATGCGGTGAACTGTGGAATAAACG |
| idnT 326F | CTGTTTAGCGAAGAGGAGATGC |
| idnT 410R | ACAAACGGCGGCGATAGC |
| hcaT 510F | GCTGCTCGGCTTTCTCATCC |
| hcaT 595R | CCAACCACGCTGACCAACC |
